# Job Satisfaction and Perceived Structural Support in Remote Working Conditions—The Role of a Sense of Community at Work

**DOI:** 10.3390/ijerph20136205

**Published:** 2023-06-22

**Authors:** Ilaria Buonomo, Bruna Ferrara, Martina Pansini, Paula Benevene

**Affiliations:** Department of Human Sciences, LUMSA University, 00193 Rome, Italy; b.ferrara@lumsa.it (B.F.); m.pansini@lumsa.it (M.P.); p.benevene@lumsa.it (P.B.)

**Keywords:** remote working, job satisfaction, sense of community, job demands, JD-R model

## Abstract

Changes in work assets due to the COVID-19 pandemic posed several challenges to employees’ well-being, especially in the light of the changes in the work organization, such as remote working and the massive use of IT. According to the literature on the role of technologies at work, the organization’s ability to support remote working is a valuable protective factor. At the same time, social distancing due to the pandemic forced employees to use a new relational asset. This, in turn, can shed new light on how the sense of connection and belonging to an organization impacts remote working experiences. This paper will test the mediational effect of structural support and sense of community at work in the link between job demands and job satisfaction in a sample of remote workers. The study involved 635 participants aged 21 to 70 (mean = 46.7, SD = 11; females = 61%). Among them, 33% had remote working experiences before the first Italian lockdown (March 2020). The research protocol included scales from the COPSOQ-III (job demands, sense of community, job satisfaction) and a questionnaire to evaluate the structural support related to the remote working asset. Results from a structural equation model showed a partial mediating effect of sense of community, but not of structural support, in the link between job demands and job satisfaction (*χ*^2^_(22)_ = 76.918, *p* = 0.00, *CFI* = 0.966, *TLI* = 0.944, *RMSEA* = 0.063 (90% CI = 0.048–0.078, *p* = 0.079), *SRMR* = 0.044). The role of such associations for future technology-based work assets is detailed in the discussion.

## 1. Introduction

The COVID-19 pandemic has significantly changed how work is conducted, impacting employees’ well-being.

Countries implemented lockdowns and social distancing measures to mitigate the virus’ spread and organizations across various sectors swiftly transitioned their operations to remote work arrangements [1]. Remote working, facilitated by the introduction of massive use of information and communication technologies (ICTs) had a peculiar effect and allowed employees to perform their duties from the safety of their homes. This shift not only ensured business continuity but also safeguarded the employees’ health and well-being [2]. The COVID-19 pandemic served as a catalyst for the realization of remote working’s potential, demonstrating its effectiveness in maintaining productivity and collaboration despite physical separation. Moreover, it unveiled the significance of digital infrastructure and flexible work arrangements in adapting to unexpected crises [3].

However, numerous studies reported mixed findings regarding the effects of remote working conditions on employees’ productivity and well-being.

On one side, remote work conditions before and during the pandemic [4,5,6] could have adverse effects on the well-being of workers, such as anxiety, depression, feelings of isolation, and a worsening of physical health [7,8,9,10,11]. Specifically, job demands and risk factors in remote work include the pressure to work outside regular hours, perceiving more intense work rhythms, feeling less autonomous, and managing technical difficulties [12]. Conversely, numerous studies have identified organizational protective factors that can prevent stress and promote workers’ well-being [13]. These protective factors include job crafting, social support, psychological safety promotion, and effective communication techniques and practices [10]. At the same time, the relational factor has played a significant role during the pandemic. Positive relations, and interpersonal trust among coworkers and supervisors had a notable influence on enhancing job satisfaction [14]. All these dimensions have proven to have an effect on employees’ overall well-being, specifically on their levels of job satisfaction [10]. On the other hand, having the opportunity to work in a safe environment and avoid contagious conditions may have influenced the employees’ job satisfaction levels with remote working [15].

Overall, given the extensive and diverse findings—both positive and negative remote working-related issues during the pandemic—a consensus has not been reached in the literature. In this regard, this study specifically focuses on the effect of remote working-related job demands on job satisfaction related to the remote work asset. By examining this specific relationship, we aim to contribute to understanding how remote work conditions during the COVID-19 pandemic influenced employees’ well-being and job satisfaction, shedding light on the role of organizational and social support to optimize remote work arrangements for their employees’ benefit.

### 1.1. Theoretical Background

Remote working, also known as teleworking or telecommuting, refers to work arrangements in which individuals perform their job activities and tasks from home or locations outside the traditional office environment [16]. Using ICTs, remote working practices are designed to allow employees greater flexibility in organizing their work schedules, eliminating the requirement for regular physical presence at a central workplace [17].

The literature shows that the association between teleworking and job satisfaction is curvilinear [8]. Specifically, it is not the remote work that per se affects the level of job satisfaction but the intensity of the remote experience [2]. Furthermore, relational aspects (such as the availability of managers’ and colleagues’ help), as well as instrumental aspects (such as training on ITC use and availability of proper ITC), are crucial for promoting employee satisfaction in remote work (e.g., [2]). More specifically, structural and social support are among the determining elements of job satisfaction (e.g., [18]). Structural support can be defined as the degree to which an organization’s policies, procedures, and practices are designed to facilitate achieving employees’ work-related goals and objectives. It encompasses the resources, support, and guidance the organization provides employees to enable them to perform their jobs effectively, efficiently, and safely. This can include but is not limited to adequate training, equipment, resources, and information, as well as a supportive work environment and effective communication channels [19].

On the other hand, the sense of community or social support is more associated with dimensions of participation and relationship. It is a shared feeling of connection, belonging, and social support among employees within a work organization. This construct encompasses the extent to which employees feel a sense of connection and shared identity with their colleagues and the degree to which they feel supported by and involved in their workplace community (e.g., [20]). Interestingly, and at the same time, the quality of work organizations and the degree of connection with colleagues and leaders were among the most crucial risk factors for job satisfaction during the pandemic (e.g., [2]).

The shift from hour-based to objective-based work has had different impacts on organizations regarding task assignment, work organization, performance assessment, and employees’ work–life balance. Research on the role of technologies at work suggests that organizations that can support remote working can better protect the well-being of their employees. For example, during the peak of the pandemic, remote working allowed employees to maintain a degree of flexibility and control over their work–life balance, which helped mitigate the pandemic’s negative effects on their mental and physical health [21].

The implications of perceived social support were similar. The social distancing measures required by the pandemic have led to changes in the relational assets of employees, highlighting the importance of a sense of connection and belonging to the organization in maintaining a positive work–life balance. Pre-pandemic previous results show that remote working leads to enhanced job satisfaction when face-to-face relationships with colleagues are maintained [22]. Regarding this, social isolation and lack of face-to-face interactions may lead to a feeling of disconnection from the organization, negatively impacting employee motivation, engagement, and job satisfaction. On the contrary, when the sense of community, participation, and cooperation is preserved even in remote working conditions, it boosts employees’ well-being and job satisfaction [23,24].

In conclusion, the changes to work assets due to the COVID-19 pandemic have shed new light on the factors that can promote and sustain the well-being of employees. Therefore, organizations that support remote working and foster a sense of connection and belonging among employees can better mitigate the negative effects of remote working assets on employees’ job satisfaction.

Overall, these studies suggest that:

**Hypothesis 1 (H1).**  *Job demands are connected to job satisfaction for remote work*.

**Hypothesis 2 (H2).**  *Structural support is connected to job satisfaction for remote work*.

**Hypothesis 3 (H3).**  *Sense of community at work is connected to job satisfaction for remote work*.

### 1.2. Conservation of Resources (COR) Theory

According to the theory of conservation of resources (COR theory; [25,26,27]), individuals strive to obtain and protect their job-related resources necessary to perform their duties while maintaining their well-being. However, the loss of resources or the threat of resource loss can lead to stress, burnout, and negative work outcomes. Furthermore, when individuals have access to renewed resources, they experience a “gain spiral”, in which positive outcomes feed on each other and lead to further gains. Thus, structural support and sense of community, as antecedents of job satisfaction, can create gain spirals that enhance employee job satisfaction. In fact, regarding structural support, when employees have access to the resources they need to reach their work goals, they can feel more supported by the organization, more effective at work, more valued in their contribution to the organizational mission, and less stressed by their work conditions [28,29]. All these beliefs and perceptions can, in turn, increase their job satisfaction, thus creating a gain spiral. As far as the sense of community at work is concerned, if employees acknowledge the existence of a valuable web of relationships at work that provides emotional support, information, and feedback as needed, this can buffer stress occurring from job demands and enhance job satisfaction, thus leading to a gain spiral (e.g., [30,31]).

These effects are available to employees involved in remote work, too. This is because gain spirals are driven by the accumulation of resources that help individuals manage job demands and control their work. Through structural support, employees may successfully cope with the challenges posed by remote work, accessing and using organizational resources.

At the same time, access to organizational resources may also fuel the gain spiral among remote workers. Some studies suggest that the relationship between structural support and employee outcomes may be even stronger for remote employees than on-site employees due to the increased importance of resources in managing the challenges of remote work (e.g., [32]). Regarding the sense of community at work, online communication channels (e.g., email, video conferencing, instant messaging) can contribute to nurturing a sense of relationship and belonging. Furthermore, it must be noted that the pandemic experience gave impulse to implementing virtual team-building activities (e.g., groups on social media platforms and gamified interactions; [33]). Research has shown that remote workers who feel a sense of community and connectedness with their colleagues are more likely to experience positive emotions, job satisfaction, and well-being (e.g., [34,35]). Overall, while the mechanisms through which the sense of community at work fosters gain spirals may differ for remote employees compared to on-site employees, the general principle still applies. Remote employees who feel a sense of community and support may be more likely to experience the gain spiral, leading to increased job satisfaction, well-being, and other positive outcomes.

Given the buffering and the gain spiral effects of both structural support and a sense of community at work, we may hypothesize the following:

**Hypothesis 4 (H4).**  *Sense of community mediates the relationship between job demands and job satisfaction for remote working*.

**Hypothesis 5 (H5).**  *Structural support mediates the relationship between job demands and job satisfaction for remote working*.

**Figure 1:**
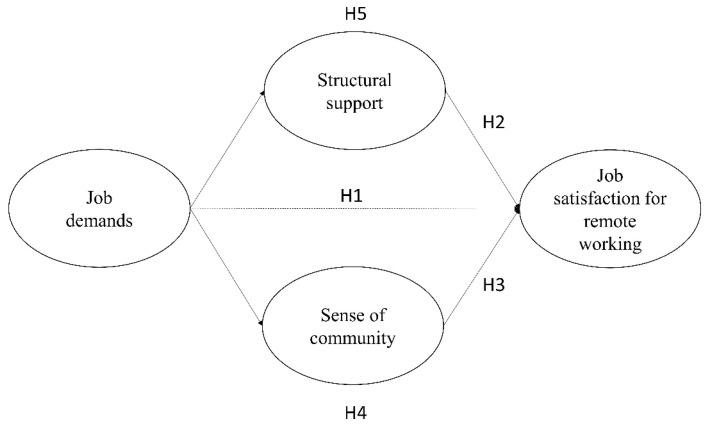
Theoretical model. Note. H4 and H5 refer, respectively, to the mediation effects of sense of community and structural support.

Figure 1 shows all the hypotheses included in the tested model.

## 2. Materials and Methods

### 2.1. Participants and Procedures

The study involved 635 participants aged 21 to 70 (mean = 46.7, SD = 11; females = 61%). Among them, 33% had remote working experiences before the first Italian lockdown (March 2020). In addition, 70% of the participants worked in organizations with more than 250 employees. Their experience in the current organization ranged from 1 to 43 years (M = 15.13, SD = 11.60). About 76% of the participants specifically worked in a service-based industry (e.g., finance, energy, legal services) and about 23% in public administration.

The employees involved in the study agreed to an informed consent form, which explained that the research team was separate from their organizations and that the information they provided would be kept confidential and anonymous. The researchers were the only ones who could access the data, and no reports would be given to management. These measures were put in place to prevent any social desirability bias.

### 2.2. Measures

A questionnaire was administered, including:

Job satisfaction for remote working—by adapting the subscale job satisfaction from the COPSOQ III to the remote work asset. The scale had 2 items, and its Cronbach’s alpha was 0.809. A sample item is: “Since you have been remote working, how much are you pleased with your job as a whole, everything taken into consideration?”.

Job demands—by adapting the subscale quantitative demands from the COPSOQ III to the remote work asset. The scale had 4 items, and its Cronbach’s alpha was 0.610. A sample item is: “Since you have been remote working, is your workload unevenly distributed, in a way that it piles up?”.

Structural support related to the remote working asset by adapting the subscale quantitative demands from the COPSOQ III to the remote work asset. The scale had 4 items, and its Cronbach’s alpha was 0.814. A sample item is: “Since you have been remote working, are you informed with reasonable advance notice about important decisions, changes, or plans at work?”.

Sense of community—using the subscale Sense of community from the COPSOQ III. The scale had 3 items, and its Cronbach’s alpha was 0.819. A sample item is: “Since you have been remote working, is there a good atmosphere between you and your colleagues?”.

### 2.3. Plan of Analyses

Firstly, we explored the data with three procedures: (a) detecting univariate and multivariate outliers using the Mahalanobis’ distance method, set to *p* < 0.001 [36]; (b) analyzing score distribution, setting skewness and kurtosis cut-off points to [−2; +2] [37]; (c) analyzing missing values and omitting them listwise [38]. After these procedures, we deleted 17 subjects and obtained the sample described in the participant section. Secondly, we tested the common method variance bias using Harman’s single-factor test. The single factor emerging from the exploratory factor analysis only accounted for 32% of the covariance among the measures, showing no issues associated with this bias [39]. Thirdly, Pearson’s correlations were measured among job demands, sense of community, job satisfaction, and structural support to verify the associations between the variables and such constructs and demographics (age, gender) and work-related variables (years of experience). After the preliminary analyses with IBM-SPSS v.24, a confirmatory factor analysis (CFA) [40] was performed to examine the measurement model with MPlus version 8 [41]. Two item parcels were created for the administered measures to enhance our model’s reliability and parsimony (JD1 and JD2 for job demands, SS1 and SS2 for structural support, JS1 and JS2 for job satisfaction for remote working). Each parcel was created by sequentially summing items assigned based on the highest to lowest item-total corrected correlations [42,43,44]. Parceling reduces free parameters to estimate and sampling error sources [42,43,44]. Because the sense of community scale includes only three items and the job satisfaction scale only two, parceling was not implemented for these scales. The robust maximum likelihood approach (MLR) was used to deal with non-normality in data [45]. To test the validity of the measures used in the model, average variance extracted (AVE) and composite reliability (CR) measures were performed (shown in Table 1). An AVE higher than 0.50 and CR higher than 0.70 indicate a good convergent validity [46]. Next, a structural equation model (SEM) [40] was implemented. Under the model, job demands were directly and indirectly (through structural support and sense of community) associated with job satisfaction for remote working. We followed a multifaceted approach to the assessment of the fit of the model [47] using the following indices: the chi-square likelihood ratio statistic, the Tucker and Lewis index (TLI), the comparative fit index (CFI), the root mean square error of approximation (RMSEA), and the standardized root mean square residual (SRMR). We accepted TLI and CFI values greater than 0.95 [48], RMSEA values lower than 0.08 [49,50], and SRMR values lower than 0.08 [48,50].

## 3. Results

Table 1 shows AVE and CR values for all the variables. According to the mentioned criteria (AVE > 0.50; CR > 0.70), the measures implemented are reliable.

Table 2 includes means, standard deviations, and correlations among all study variables.

**Figure 2:**
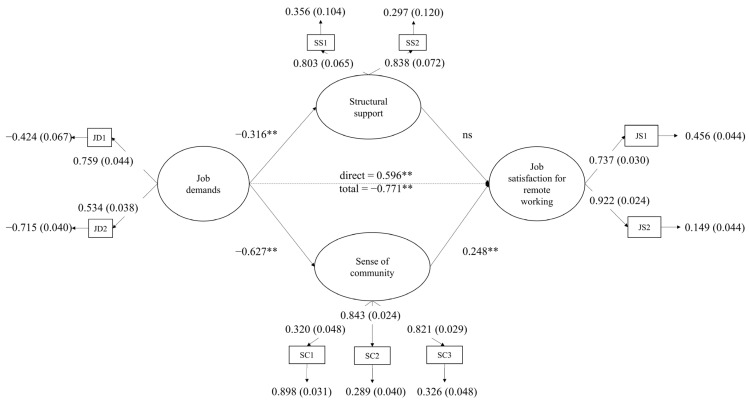
Final model. Results of the structural equation model. Standardized effects and significance values are reported. Note. ** = *p* < 0.01, ns—not significant. JD—job demands; SC—sense of community; JS—job satisfaction for remote working; SS—structural support.

Results from the structural equation model (Figure 2) showed a partial mediating effect of sense of community, but not of structural support, in the link between job demands and job satisfaction (χ^2^_(22)_ = 76.918, *p* = 0.00, CFI = 0.966, TLI = 0.944, RMSEA = 0.063 (90% CI = 0.048–0.078, *p* = 0.079), SRMR = 0.044).

All the mentioned hypotheses were confirmed, except H2 (structural support is associated with job satisfaction for remote working) and H5 (structural support mediates the relationship between job demands and job satisfaction for remote working). Job satisfaction for remote working was significantly associated with job demands (H1; *β_direct_* = −0.596, *p* < 0.01; *β_total_* = −0.771, *p* < 0.01) and sense of community (H3; *β* = 0.248, *p* < 0.01).

## 4. Discussion

Overall, our results have shown partial mediation of the sense of community but not structural support in the relationship between job demands and job satisfaction.

For the results concerning the sense of community, the model confirms that relationships can be a resource that triggers a gain spiral in the organizational context, even at a distance. The accumulation of resources, in turn, can reduce the adverse effects of job demands, for example, by making relationship-based coping mechanisms more available. Recently linked with the COR theory, the crossover effect suggests that working together influences each other’s mental states, constructing a shared reality at work [26,51,52]. Our findings suggest that the principles and applications of the COR theory at work still work in the virtual context. Thus, it is possible that having the opportunity to actively participate in planning, problem-solving, and decision-making processes online, and doing so in a positive relational climate, contributes to creating a sense of job satisfaction for remote workers. In this regard, our results are consistent with one longitudinal study carried out during the COVID-19 pandemic from 2020 to 2021, in which remote workers’ sense of community and perceived social support led to increased self-rated health in employees [53]. Previous studies found a significant relationship between perceived social support and working outcomes related to employees’ well-being and job satisfaction [21]. Before the COVID-19 pandemic, research showed that social support had a key role in the adjustment of employees to remote working and positively influenced the effectiveness in which remote working was implemented and employees’ job satisfaction [18]. Such mechanisms become more relevant in times of change. During the pandemic, social support from superiors and colleagues involved the creation of a shared identity and a sense of mutuality between organizational members and preserving a caring and safe environment, allowing them to pursue objectives at work [54]. In addition, the quantity and quality of social communication are crucial when employees work remotely. As shown by Fonner and Roloff [8], employees are more satisfied if they frequently communicate with supervisors and perceive to be involved in the exchange of high-quality and timely information. Job resources such as social support from coworkers and supervisors, participation in the decision-making process, and timely feedback on activities and results may act as protective factors and enhance the well-being at work in employees [55].

Regarding the lack of a mediation effect of structural support, this unexpected result contrasts with the conservation of resources (COR) theory, which suggests that even material resources could trigger a gain spiral. Furthermore, this contrasts with other studies (e.g., [56]) in which organizational support positively mediates the relationship between job demands and employees’ strain outcomes. Supportive practices such as technical support from IT professionals or training designed to enhance skills for new technology may reduce employees’ ICT-related stress symptoms and enable them to feel more effective in performing their tasks. However, this could mean that receiving technical and communication organizational support can cause employee stress levels to decrease while not increasing job satisfaction. Studies on well-being and satisfaction showed that positive and negative emotions are independent, both from a phenomenological and a biological point of view [57,58]. Thus, it is not surprising that feeling instrumentally supported at work can reduce stressful feelings and related thoughts while not influencing job satisfaction.

One possible explanation for the results obtained in this study is the salience of instrumental support. According to Dahlstrom [59], in conditions of low or no face-to-face interaction, organizational measures focused on relationships are more effective in promoting job satisfaction and commitment compared to those focused on task orientation. This is especially true in private organizations where the structuring and formalization of procedures are lower than those of public organizations. Another element concerns the quality of instrumental support. It is known that the quality of tools and infrastructures to support remote work has evolved dramatically in recent years, especially in response to the needs arising from the COVID-19 pandemic. For example, an OECD report in 2021 showed that France, Japan, Brazil, and Australia doubled the incidence of remote working between 2019 and 2020, while in Italy, where this study was conducted, it quadrupled. In this regard, the Italian work context has been characterized by specific critical issues about the organizations’ ability to provide instrumental support. A qualitative study on HR managers from different organizations shows challenging aspects. First, due to the imposed and unexpected shift to remote working, employees were not prepared to deal with the new way of communication, both from a technical and social perspective. As a result, the need emerged to develop new competences and new professional figures. Secondly, the need to review performance measurement systems and incentive and reward procedures and establish new habits to foster a safe and caring work environment, even at a distance [60].

It is, therefore, plausible that repeating this study after some time, under conditions of greater stability in the tools and infrastructures to support remote work, could provide new results that are more consistent with the hypotheses formulated by the COR theory.

## 5. Conclusions

The results regarding the positive effect of the sense of community on the relationship between job demands and satisfaction with the remote working experience suggest the importance of promoting trust and a sense of belonging for remote workers. When remote workers perceive a strong sense of community within their work environment, it can act as a buffer against the potentially negative effects of high job demands. This implies that organizations should prioritize efforts to promote trust-building activities and create a supportive work culture for remote employees.

Given the heterogeneity of the dimensions and effects of the sense of community, future studies may further explore the specific dimensions of the sense of community in the workplace by utilizing more comprehensive tools that capture differential effects. Regarding the lack of mediation effect of structural support, our findings may have been influenced by the specific historical data collection period. Future studies may explore the role of organizational support under conditions of greater infrastructure stability and by comparing different types of organizations.

## 6. Practical Implications

Results show that fostering trust and a feeling of belonging among remote employees play a crucial role in the online context. In this regard, it is interesting to consider the applicability of compassionate leadership training to the remote work environment. Compassionate leadership is a form of leadership that uses the relationship with others as a tool to achieve two outcomes: (1) greater employee participation and engagement in identifying measures to support performance and well-being; (2) the creation of a climate of trust and mutual, non-judgmental listening. Compassionate leaders act as relationship models and value everyone’s experiences, generating a compassionate organizational culture. In this context, it is interesting to consider the experience of Kotera et al. [61] regarding online morning huddles, i.e., informal meetings in virtual teams aimed at sharing work and daily life experiences and discussing each other’s well-being outside the work context.

An additional aspect of interest concerns the construction, through relationships, of a shared horizon of meaning at work. In 1974, Sarason [62] defined the sense of community at work as the outcome of a process in which work is one of the employee’s referents to structure and gives meaning to their lives and the values and qualities they recognize. All forms of participation and listening can favor this type of work experience, providing moments of sharing and self-evaluation (e.g., regarding the alignment between personal and organizational goals). Useful tools for this purpose include establishing communication channels easily usable by managers and middle managers, unambiguous and aligned with offline channels, using surveys, supervision meetings, or evaluation questionnaires, and sharing results of data collected through the channels mentioned above with all employees. Such initiatives can enhance job satisfaction and contribute to the overall well-being and productivity of remote workers in their virtual work settings.

## 7. Limits

Despite the valuable insights that our study has provided, it is important to acknowledge its limitations. Firstly, the data collection was carried out during the COVID-19 pandemic, which could have impacted the participants’ responses. While we tried to mitigate this bias by asking questions about work and well-being in general, we cannot rule out the possibility that the pandemic has influenced the responses unexpectedly. Secondly, our study design was cross-sectional, so we could not establish a causal relationship between the variables. Future research could benefit from a longitudinal design to track changes in work and well-being over time. Finally, our study relied solely on quantitative data, which may not capture the nuances of the participants’ experiences and perspectives. Incorporating qualitative methods in future research could provide a more in-depth understanding of the factors that shape work experiences and job satisfaction.

## Figures and Tables

**Table 1:** AVE and CR values for all the variables in the study.

	AVE	CR
Engagement	0.610	0.819
Experienced compassion	0.613	0.823
Ethical leadership	0.895	0.962
Subjective Well-being	0.755	0.860

Note. AVE—average variance extracted; CR—composite reliability.

**Table 2:** Descriptive statistics (M—mean; SD—standard deviation) and correlations.

Variables	Descriptives		Correlations			
	M	SD	Job Demands	Sense of Community	Structural Support	Job Satisfaction for Remote Working
Job demands	2.40	1.05	-	−0.478 **	−0.250 **	−0.640 **
Sense of community	3.05	0.80		-	0.285 **	0.511 **
Structural support	2.63	0.97			-	0.211 **
Job satisfaction for remote working	3.39	1.12				-

** = *p* < 0.01.

## Data Availability

Data are available upon request.
